# Age Dependent Dysfunction of Mitochondrial and ROS Metabolism Induced by Mitonuclear Mismatch

**DOI:** 10.3389/fgene.2019.00130

**Published:** 2019-02-20

**Authors:** Nicolas Pichaud, Roxanne Bérubé, Geneviève Côté, Claude Belzile, France Dufresne, Geneviève Morrow, Robert M. Tanguay, David M. Rand, Pierre U. Blier

**Affiliations:** ^1^Laboratory of Comparative Biochemistry and Physiology, Department of Chemistry and Biochemistry, Université de Moncton, Moncton, NB, Canada; ^2^Laboratoire de Physiologie Animale Intégrative, Département de Biologie, Université du Québec à Rimouski, Rimouski, QC, Canada; ^3^Institut des Sciences de la mer de Rimouski, Université du Québec à Rimouski, Rimouski, QC, Canada; ^4^Laboratoire d’Écologie Moléculaire, Département de Biologie, Université du Québec à Rimouski, Rimouski, QC, Canada; ^5^Laboratoire de Génétique Cellulaire et Développementale, Département de Biologie Moléculaire, Biochimie Médicale et Pathologie, Université Laval, Quebec City, QC, Canada; ^6^Department of Ecology and Evolutionary Biology, Brown University, Providence, RI, United States

**Keywords:** aging, *Drosophila*, mitochondrial respiration, mitonuclear incompatibility, reactive oxygen species, replication, tRNA

## Abstract

Mitochondrial and nuclear genomes have to coevolve to ensure the proper functioning of the different mitochondrial complexes that are assembled from peptides encoded by both genomes. Mismatch between these genomes is believed to be strongly selected against due to the consequent impairments of mitochondrial functions and induction of oxidative stress. Here, we used a *Drosophila* model harboring an incompatibility between a mitochondrial tRNA^tyr^ and its nuclear-encoded mitochondrial tyrosine synthetase to assess the cellular mechanisms affected by this incompatibility and to test the relative contribution of mitonuclear interactions and aging on the expression of impaired phenotypes. Our results show that the mitochondrial tRNA mutation caused a decrease in mitochondrial oxygen consumption in the incompatible nuclear background but no effect with the compatible nuclear background. Mitochondrial DNA copy number increased in the incompatible genotype but that increase failed to rescue mitochondrial functions. The flies harboring mismatch between nuclear and mitochondrial genomes had almost three times the relative mtDNA copy number and fifty percent higher rate of hydrogen peroxide production compared to other genome combinations at 25 days of age. We also found that aging exacerbated the mitochondrial dysfunctions. Our results reveal the tight interactions linking mitonuclear mismatch to mitochondrial dysfunction, mitochondrial DNA regulation, ROS production and aging.

## Introduction

Dysfunctional mitochondria are thought to be a proximal mechanism for aging due to the dual role of mitochondria as a source and target of reactive oxygen species (ROS) associated with aging (Harman, 1972; Horan et al., 2012; Yun and Finkel, 2014). In mitochondria, the oxidative phosphorylation (OXPHOS) process requires the proper assembly and function of the electron transport system (ETS) (Blier et al., 2001). The ETS consists of different complexes encoded by both the mitochondrial and the nuclear genomes (mtDNA and nuDNA, respectively) directly participating in electron transport and proton pumping (complexes I, III and IV) whilst complex V uses the created proton gradient to regenerate ATP. Other exclusively nuclear-encoded enzymatic complexes, such as complex II and glycerol-3-phosphate dehydrogenase (mG3PDH), are part of this system and contribute to increase the flux of electron into the ETS without individually pumping additional protons. The ETS is therefore a complex system and its integrity is achieved and maintained by a tight coordination between the nuclear and the mitochondrial encoded peptides (Blier et al., 2001; Wolff et al., 2014). The mitochondrial ETS components are also major ROS producers in cells (Brand, 2010; Andreyev et al., 2015). Although harmful to macromolecules and believed to elicit oxidative stress associated to age-related diseases, ROS are also important messengers inciting a retrograde (mitochondria to nucleus) response and thus modulate mitochondrial content and functions according to the cell requirements (Yun and Finkel, 2014; Shadel and Horvath, 2015). It is also believed that replication errors, such as deletion of mtDNA are a major force driving aging and age-related diseases (Bai and Wong, 2005; Clay Montier et al., 2009; Stumpf and Copeland, 2011; DeBalsi et al., 2017; Kauppila et al., 2017).

It has been suggested that long-lived species do not tolerate mismatch between nuclear and mitochondrial genomes because of the importance of fine tuning aerobic metabolism and the role of ROS generation (and buffering) in the induction of apoptosis (Lane, 2011). The nature and properties of mtDNA (small effective population size, lack of recombination and high mutation rate) makes it prone to accumulate deleterious mutations that have been associated with a plethora of disease phenotypes (Horan et al., 2013; Wolff et al., 2014). At the level of population or species, compensatory selective changes may arise in the nuDNA to cope for the potential adverse effects caused by deleterious mtDNA mutations and to restore mitochondrial functions (Osada and Akashi, 2012; Horan et al., 2013; Ballard and Pichaud, 2014; Wolff et al., 2014). Moreno-Loshuertos et al. (2011) found that mouse cells expressing a mitochondrial tRNA mutation have a significant OXPHOS deficiency that is compensated by enhanced mitochondrial biogenesis. In some cases, the nuclear genome may fail to stage compensatory responses to deleterious effects of mtDNA mutations and can even trigger the associated disease phenotype. Potluri et al. (2009) found that a disease phenotype -an encephalomyopathy characterized by a complex I deficiency- only manifests once the mtDNA polymorphism is expressed alongside a specific nuDNA variant. Functional incompatibility may thus arise from the combination of mtDNA and nuDNA variants that will cause life-threatening dysfunctions at older ages (Wallace, 2010). Mitonuclear disruption via experimental hybridization has also been shown to induce genotypic incompatibility leading to decreased fitness and mitochondrial defective phenotype in several species (Sackton et al., 2003; Rand et al., 2004; Ellison and Burton, 2008; Dobler et al., 2014). In invertebrates such as Drosophila and Tigriopus, the detrimental effects might be negligible in closely related populations (Pichaud et al., 2012) and increase among more divergent populations of the same species (Rand, 2001; Burton et al., 2007; Meiklejohn et al., 2013). They can cause even more drastic consequences in distantly related taxa, including in mammals (Barrientos et al., 1998; McKenzie et al., 2003). The cellular cascade behind mitonuclear incompatibility and the compensatory mechanisms allowing restoration of adapted phenotypes are still relatively unknown in animals. This is mainly due to a lack of relevant models in which these incompatibilities have been thoroughly characterized.

In this study, we took advantage of the generation of mtDNA-nuDNA combinations from two related *Drosophila* species. Specifically, mtDNAs from either *D. melanogaster* (*ore*) or *D. simulans* (*simw^501^*) were substituted into two *D. melanogaster* wild-type nuclear backgrounds, *OreR* and *Aut* (Montooth et al., 2010; Meiklejohn et al., 2013), which generated four different mtDNA-nuDNA combinations. The (*simw^501^*) mitochondrial genome differs from the (*ore*) at the level of a tRNA^tyr^ single nucleotide polymorphism (SNP), whereas the *Aut* nuDNA differs from the *OreR* by a SNP in a mitochondrial tyrosine synthetase. The (*simw^501^*);*OreR* combination is characterized by multiple deleterious effects on development, reproduction, locomotion, respiration of isolated mitochondria and mitochondrial morphology due to an incompatibility between (*simw^501^*) mitochondrial tRNA^tyr^ and the *OreR* nuclear-encoded mitochondrial tyrosine synthetase (Meiklejohn et al., 2013; Holmbeck et al., 2015; Zhang et al., 2017). These mitonuclear genotypes are therefore a good model to investigate the contribution of mtDNA, nuDNA and mitonuclear interactions in the cellular mechanism(s) affecting aging and fitness, as well as their impact(s) on sensitive mitochondrial functions and how this translates in terms of ROS management and mitochondrial DNA regulation. Other studies have shown the effect of mtDNA divergences on mitochondrial bioenergetics in Drosophila (Correa et al., 2012; Pichaud et al., 2012, 2013). Notably, Correa et al. (2012) showed that a small set of mtDNA mutations (in the ND2 subunit of complex I, in a tRNA and in a 12S rRNA) were associated to mitochondrial dysfunctions as the organism aged (Correa et al., 2012). In our study, we compared the metabolic and cellular phenotypes of Drosophila sampled at different ages harboring either normal epistatic mitonuclear interaction or single mutations in both nuclear and mitochondrial genomes resulting in breakdown of mitonuclear co-adapted genes. Thus, this model allows us to evaluate how mitonuclear mismatch due to point mutations is associated to mitochondrial alterations and estimate to which extent organism can survive disruption from mitonuclear mismatch, giving insights into the underlying mechanisms of genome co-evolution. We hypothesized that the mitonuclear incompatibility translates into mitochondrial dysfunctions, and ROS overproduction potentially leading to oxidative stress and premature aging. To test this hypothesis, we measured *in situ* mitochondrial respiration, mitochondrial content, mtDNA copy number, oxidation by ROS, H_2_O_2_ production as well as oxidative damages to proteins and lipids in the four mitonuclear genotypes sampled at 15 and 25 days of age.

## Materials and Methods

### Fly Maintenance and Experimental Design

Drosophila lines were provided by D. Rand and were constructed by introgressing mitochondrial genomes i.e., (*ore*) from *D. melanogaster* or (*simw^501^*) from *D. simulans si*II haplotypes, and by replacing the nuclear genomes with either *OreR* or *Aut* chromosomes using non-recombining balancer chromosomes, and were the same as previously studied by others (Montooth et al., 2010; Meiklejohn et al., 2013). These lines are available upon request. The genotypes constructed by these crosses combine polymorphisms in mtDNA and nuclear chromosomes that generate significant mitochondrial-nuclear epistasis for fitness (Meiklejohn et al., 2013). All lines were maintained on standard cornmeal medium at constant temperature (24.0 ± 0.1°C), humidity (50% RH), diurnal cycle (12 h:12 h light:dark) and density (approximately 50 flies for 25 ml of standard cornmeal medium). Only males were studied because mitochondrial dysfunction is hypothesized to be more pronounced in males than females (Gemmell et al., 2004; Innocenti et al., 2011; Camus et al., 2012). We sampled the four mitonuclear genotypes at two different ages (15 and 25 days old). Although 25 days old do not represent advanced age in Drosophila, females of the (*simw^501^*);*OreR* mitonuclear genotype harboring the incompatibility have a mean lifespan as low as 32 days depending on the diet (Zhu et al., 2014). Since the male-specific mitochondrial mutation load in *D. melanogaster* is more pronounced and results in decreased longevity and increased rate of senescence (Camus et al., 2012), we selected 25 days old as a representative age to determine the occurrence of premature aging. For each treatment (mitonuclear genotype × age), twelve different pools of 3 flies were used (*n* = 12) for all the experiments except for the measurement of H_2_O_2_ production in isolated mitochondria in which six different pools of 30 flies were used (*n* = 6). On each experimental day, thoraces were dissected and either directly processed for mitochondrial isolation, high-resolution respirometry, and confocal microscopy or immediately stored at -80°C for DNA extraction and biochemical analysis.

### DNA Sequencing

Genomic DNA was extracted from 3 thoraces using E.Z.N.A.^®^ Tissue DNA kit according to the manufacturer’s instructions. Mitochondrial and nuclear genomic DNA were sequenced by amplifying a 948 bp region of the mitochondrial large ribosomal RNA gene and a 1991 bp fragment of the 215 kDa subunit of RNA polymerase II, respectively. Each 25 μl total reaction included 2.5 μl of 10X buffer, 2.2 mM of MgCl_2_, 0.2 mM of dNTPs mix, 2.5 μM of each forward (GAAATGAAATGTTATTCGTTTTTAAAGGTATCTAG for mtDNA gene and CGGGTGGAGAGAAGTATCGC for nuDNA gene) and reverse (AGAAACCAACCTGGCTTACACCGGTTTGAACTCAG for mtDNA gene and GGCTATGGAGTCGGTGATGG for nuDNA gene) primers, 0.3 U of Taq polymerase and 30 ng of DNA. Each DNA fragment was verified on 1.5% agarose gel. Cleaning and sequencing of PCR products were conducted at McGill University and Génome Québec Innovation Centre (QC, Canada). All DNA sequences were then analyzed in Sequencher^®^ version 4.9 sequence analysis software.

Sequences obtained from the mitochondrial and nuclear genomic DNA of the different genotypes were aligned using MEGA version 5.1 (Tamura et al., 2011). Each DNA sequence was blasted against GenBank database (Megablast algorithm, Morgulis et al., 2008) and showed that the different lines harbored the expected divergences and the mtDNA and nuDNA specific to each genotype. All sequences are available in GenBank (KR231637 to KR231660).

### Relative Mitochondrial DNA Copy Number

The relative mtDNA copy number per diploid nuclear nuDNA genome was evaluated as previously described (Correa et al., 2012). Mitochondrial and nuclear genomic DNA were quantified by amplifying a 102 bp region of the mitochondrial large ribosomal RNA gene and a 221 bp region of the 215 kDa subunit of RNA polymerase II, respectively. Each reaction included 7.5 μL of SensiFAST SYBR^®^ No-ROX mix, 1 ng of DNA, 0.2 μM of each forward (CAACCATTCATTCCAGCCTTC for mtDNA gene and AGGCGTTTGAGTGGTTGG for nuDNA gene) and reverse (GTCTAACCTGCCCACTGAAA for mtDNA gene and CGCTTTGGGCTTTTTGGAT for nuDNA gene) primers. Reactions were run in triplicates on a LightCycler^®^ 480 Real-Time PCR System using the following thermal profile: 95.0°C for 3 s, followed by 40 cycles of 95.0°C for 30 s, 60.0°C for 30 s, and 72°C for 30 s. The efficiency of each primer set (E_nu_ and E_mt_) was determined using appropriate serial dilutions before sample analyses and primer specificity for each gene was verified by regular PCR and dissociation curve analysis following qPCR protocols. Copy number of the mtDNA gene relative to the nuDNA gene was calculated using the cycle thresholds (Ct) for each gene and kinetic PCR efficiency correction with the following formula: 2^∗^(E_nu_^Ctnu^/E_mt_^Ctmt^).

### Fiber Permeabilization and High-Resolution Respirometry

Thorax muscles (3 thorax for each measurement) were permeabilized at 4°C using BIOPS relaxing solution (Kuznetsov et al., 2008) complemented with saponin as previously described for *Drosophila* (Pichaud et al., 2011; Simard et al., 2018). They were then blotted, weighed and transferred into an Oxygraph-2k respirometer (Oroboros Instruments, Innsbruck, Austria) calibrated with air-saturated respiration medium at 24°C containing 115 mM KCl, 10 mM KH_2_PO_4_, 2 mM MgCl_2_, 3 mM HEPES, 1 mM EGTA, 0.2% BSA, pH 7.2. All measurements were expressed as means of respiration rates expressed in pmol of oxygen consumed per second per mg of permeabilized fibers ± s.e.m and are presented with the abbreviation(s) of the complex(es) involved followed by the state of respiration (complex-STATE) as previously described (Pichaud et al., 2013; Simard et al., 2018). After monitoring CI-LEAK with pyruvate, proline, and malate, sequential injections of different compounds were performed in the following order: excess ADP (5 mM) to measure CI-OXPHOS; cytochrome c (15 μM) allowing the evaluation of the functional integrity of the outer mitochondrial membrane; glycerol-3-phosphate (20 mM) to monitor maximum OXPHOS with the contribution of complex I and glycerol-3-phosphate dehydrogenase (CI+mG3PDH-OXPHOS); and FCCP (optimum concentration reached between 0.75 and 1.25 μM) to stimulate uncoupled respiration for complex I and mG3PDH as a measure of ETS capacity (CI+mG3PDH-ETS). Rotenone (1 μM) and antimycin A (2.5 μM) were then injected to inhibit complexes I and III, respectively, and measure the residual oxygen consumption which was subtracted from the other rates. Finally, N,N,N′,N′-tetramethyl-p-phenylenediamine (TMPD) and ascorbate (0.5 μM and 2 mM, respectively) were added to measure complex IV activity which was corrected from chemical background after complete inhibition with sodium azide. From this mitochondrial respiration rates, the P/L ratio (P/L = CI-OXPHOS/CI-LEAK) was calculated to evaluate mitochondrial quality, and increase of oxygen flux after injection of cytochrome c was used to determine the integrity of the outer mitochondrial membrane (Gnaiger, 2009; Lemieux et al., 2017).

### Image Capture and Analysis of Mitochondrial Content and Oxidative Stress Detection

Single muscle fibers from thorax were dissected in BIOPS relaxing solution (Kuznetsov et al., 2008) and incubated with 100 nM of Mitotracker^®^ Green FM and 5 μM of CellROX^®^ Deep Red Reagent (Molecular Probes, Inc., Eugene, OR, United States) for 30 min at 24°C in order to label mitochondria and oxidation by ROS, respectively. Labeled fibers were then observed with a Zeiss LSM 700 confocal laser scanning microscope coupled to an Axio Observer inverted microscope (Carl Zeiss Canada Ltd., Toronto, ON, Canada). Images were acquired in 8 bits using LD Plan-Neofluar 40x/0.6 Korr M27 objective and captured using ZEN 2012 1.1.1.0 (Carl Zeiss Canada Ltd., Toronto, ON, Canada). Excitation/emission wavelength were λ_exc_/λ_em_ = 488/BP 420–550 nm and λ_exc_/λ_em_ = 639/LP 640 nm for Mitotracker^®^ Green FM and CellROX^®^ Deep Red Reagent, respectively. 20 z-stack confocal images (in *x*/*y* axis, 31.2 × 31.2 μm^2^ size) of fibers were acquired using z-slices of 0.91 – 1.79 μm with pinhole size of 70.14 μm. Image J software (National Institutes of Health) was used to analyze and quantify fluorescence in confocal images. For quantification, z-projections of 10 raw confocal images were generated and were processed using the “Autothreshold Default” method of Image J. Fluorescence measurements in the resulting images were performed using the “Integrated Density” option [which corresponds to the mean fluorescence value in the region of interest (ROI) multiplied by the ROI area] and were corrected with background readings. Results are expressed as arbitrary units of fluorescence intensity.

### Mitochondrial Isolation and H_2_O_2_ Production

Intact mitochondria were isolated at 4°C as previously described (Pichaud et al., 2010) using 30 thoraces. The hydrogen peroxide production rate was determined using Amplex^®^ red reagent (Invitrogen) and an EnVision^®^ microplate reader (PerkinElmer, Waltham, MA, United States) set at 24°C with excitation/emission set at 560/587 nm. For each sample, maximal H_2_O_2_ production at the level of complex I (i.e., in presence of pyruvate, malate, proline, ADP, G3P, and rotenone), as well as at the level of complexes I and III (i.e., in presence of pyruvate, malate, proline, ADP, G3P, rotenone, and antimycin A) were measured. Results are presented as means ± s.e.m. of nmol H_2_O_2_ produced per minute per mg of proteins.

### Enzymatic Analysis and Markers of Oxidative Stress

Activity of NADH:ubiquinone oxidoreductase (complex I) and ubiquinone:cytochrome c oxidoreductase (complex III), CI+CIII, as well as citrate synthase (CS) activity were evaluated in crude homogenates of thorax (pool of 3 flies) using an EnVision^®^ microplate reader (PerkinElmer, Waltham, MA, United States) set at 24°C as previously described (Pichaud et al., 2010). CI+CIII activity was measured by following the reduction of iodonitrotetrazolium (INT) at 490 nm for 4 min using a 100 mM potassium phosphate, 0.85 mM NADH, 2 mM INT, 0.03% (v/v) triton X-100, pH 8.5 reaction medium. CS activity was measured as a marker of mitochondrial content (Larsen et al., 2012) by following the reduction of 5,5-dithiobis-2-nitrobenzoic acid (DTNB) at 412 nm using a 100 mM imidazole-HCl buffer containing 0.1 mM DTNB, 0.1 mM acetyl-CoA, and 0.15 mM oxaloacetic acid (omitted from the blank), pH 8.0. Enzymatic activities were normalized by total protein content measured using the bicinchoninic acid method (Smith et al., 1985) and are expressed as U.mg^-1^ proteins. The same homogenates were used to evaluate oxidative damages to proteins (protein carbonyls) and lipids (thiobarbituric acid reactive substances, TBars) using commercially available kits from Cayman Chemicals (Ann Arbor, MI, United States) and following the manufacturer’s protocol.

### Statistical Analysis

All statistical analyses were performed with the R software (Free Software Foundation; Boston, MA, United States). For all parameters, three-way ANOVAs considering MtDNA, NuDNA and Age as fixed factors were performed. When an interaction (MtDNA × NuDNA, MtDNA × Age, NuDNA × Age, or MtDNA × NuDNA × Age) revealed a significant effect, multiple comparisons were tested with pairwise comparisons of the least-squares means using adjusted *P*-values (Tukey method) with significance set at *P*<0.05. Normality and homogeneity of variance were verified beforehand using Shapiro–Wilk and Levene’s tests, respectively, and data were ln-transformed when required. *F*-ratios and significance from three-way ANOVAs on all the different parameters studied are presented in Table 1.

**Table 1:** Results of three-way ANOVAs showing F ratios for MtDNA, NuDNA, Age and their interactions.

	Error *df*	MtDNA *df* = 1	NuDNA *df* = 1	Age *df* = 1	MtDNA × NuDNA *df* = 1	MtDNA × Age *df* = 1	NuDNA × Age *df* = 1	MtDNA × NuDNA × Age *df* = 1
**Mass-specific O_2_ fluxes**								
CI-LEAK	88	26.87^∗∗∗^	0.51	6.04^∗^	1.20	4.37^∗^	1.52	1.56
CI-OXPHOS	88	44.69^∗∗∗^	0.73	6.97^∗∗^	0.49	1.61	0.23	0.004
P/L ratio	88	0.67	3.54	13.53^∗∗∗^	1.49	0.02	1.87	0.01
CI+mG3PDH-OXPHOS	88	5.81^∗^	8.89^∗∗^	0.40	9.33^∗∗^	0.04	0.41	3.19
CI+mG3PDH-ETS	88	4.81^∗^	9.64^∗∗^	0.12	9.77^∗∗^	0.13	0.51	3.18
Complex IV	88	3.51	19.24^∗∗∗^	0.83	0.63	0.30	0.03	0.73
**Enzymatic activity**								
CI+CIII	88	24.29^∗∗∗^	22.606^∗∗∗^	2.29	14.63^∗∗∗^	0.08	0.14	0.12
Citrate synthase	88	36.17^∗∗∗^	10.61^∗∗^	0.27	10.07^∗∗^	2.83	5.51^∗^	0.004
**Confocal microscopy**								
Mitochondrial content	88	46.39^∗∗∗^	11.83^∗∗∗^	1.55	10.65^∗∗^	0.09	3.13	1.71
ROS content	88	8.37^∗∗^	2.71	2.75	2.33	0.002	0.00	0.94
**Maximal H_2_O_2_ production**								
Complex I	44	20.04^∗∗∗^	14.58^∗∗^	33.89^∗∗∗^	10.83^∗∗^	8.07^∗∗^	0.15	0.22
Complexes I+III	44	42.97^∗∗∗^	14.73^∗∗^	46.61^∗∗∗^	23.25^∗∗∗^	20.44^∗∗∗^	2.36	4.57^∗^
**Mitochondrial replication**								
MtDNA copy number	88	65.50^∗∗∗^	74.59^∗∗∗^	6.08^∗^	69.66^∗∗∗^	0.57	0.10	0.004
**Oxidative damages**	
TBars	88	5.02^∗^	8.38^∗∗^	23.26^∗∗∗^	2.63	0.18	0.17	0.001
Carbonyls	88	1.48	1.35	0.78	0.67	0.67	1.11	0.25

*^∗^P<0.05, ^∗∗^P<0.01, and ^∗∗∗^P<0.001.*

## Results

### Relative MtDNA Copy Number and Mitochondrial Content

**FIGURE 1:**
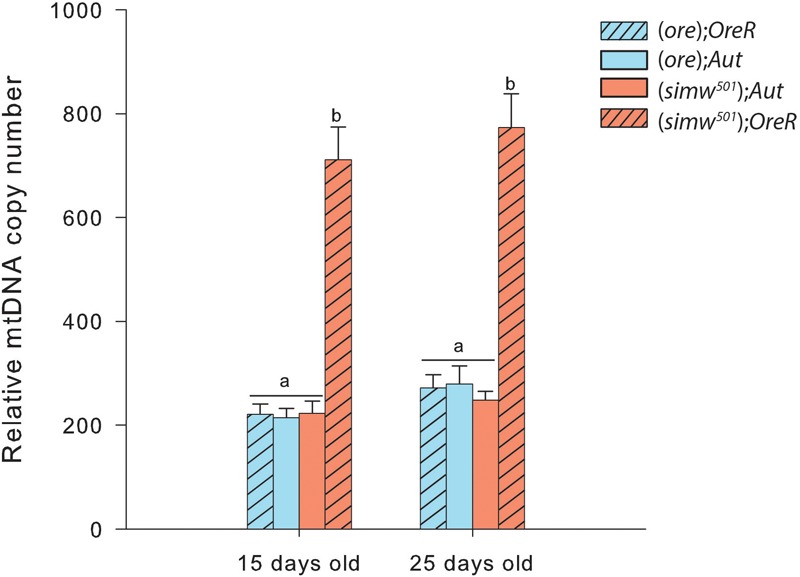
Relative mitochondrial DNA copy number in thorax of *Drosophila*. Specific differences between the four genotype combinations are denoted with letters.

**FIGURE 2:**
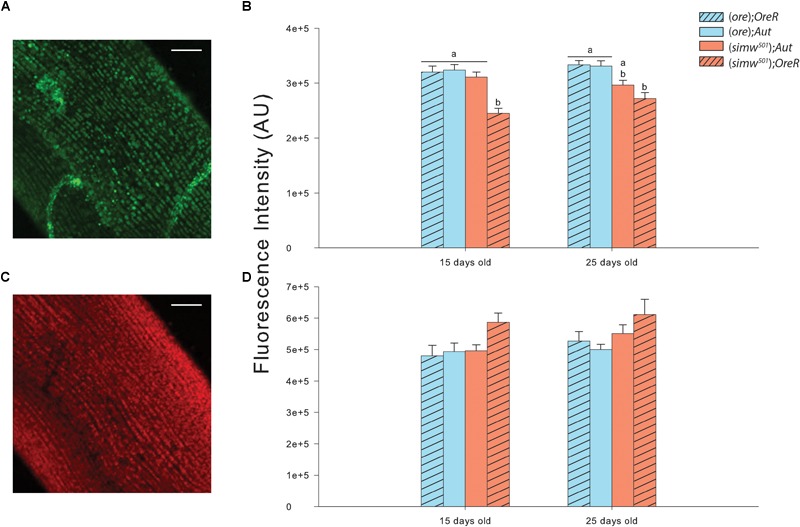
Mitochondrial content and oxidation by ROS measured in thorax muscles of *Drosophila*. **(A)** Measurements of mitochondrial content from (*ore*)*;OreR* flies of 15 days old using Mitotracker^®^ Green FM. The picture is the sum of 20 z-stack confocal images. Scale bars: 25 μm. **(B)** Fluorescence intensity measured in thorax muscles at 15 and 25 days old for mitochondrial content with Mitotracker^®^ Green FM. Results are expressed as arbitrary units (AU) of fluorescence intensity measured using the integrated density option of Image J software in 10 raw confocal images. All images were corrected with background readings. Letters denote differences between the four genotype combinations (*post hoc* Tukey’s test). **(C)** Measurements of ROS content from (*ore*)*;OreR* flies of 15 days old using CellROX^®^ Deep Red Reagent. The picture is the sum of 20 z-stack confocal images. Scale bars: 25 μm. **(D)** Fluorescence intensity measured in thorax muscles at 15 and 25 days old for ROS content with CellROX^®^ Deep Red Reagent. Results are expressed as AU of fluorescence intensity measured using the integrated density option of Image J software in 10 raw confocal images. All images were corrected with background readings. Letters denote differences between the four genotype combinations.

MtDNA copy number was influenced by MtDNA, NuDNA, Age and the MtDNA × NuDNA interaction (Table 1). Specifically, (*simw^501^*);*OreR* flies had an almost three-fold increase in mtDNA copy number compared to (*ore*)*;Aut*, (*ore*)*;OreR*, and (*simw^501^*);*Aut* when evaluated at both 15 and 25 days old (all *P*-values < 0.001, Figure 1). We also evaluated the mitochondrial content of muscle by measuring the fluorescence intensity of the mitochondrial marker Mitotracker^®^ Green FM (Figure 2A). The MtDNA, the NuDNA as well as the interaction MtDNA × NuDNA had strong effects on mitochondrial content (Table 1). Surprisingly, mitochondrial content was significantly lower in (*simw^501^*);*OreR* compared to the other lines at 15 days old (all *P*-values < 0.001, Figure 2B), and at 25 days old when compared to (*ore*);*OreR* and (*ore*);*Aut*. To validate these results obtained with confocal microscopy, we also evaluated the CS activity as a proxy of mitochondrial content (Larsen et al., 2012). The CS activity was also influenced by the MtDNA, the NuDNA as well as the interaction MtDNA × NuDNA (Table 1). Moreover, CS activity was significantly decreased in (*simw^501^*);*OreR* compared to the other lines at 15 days old (all *P*-values < 0.001, Table 2) and at 25 days old when compared to (*ore*);*OreR* (*P* = 0.008, Table 2).

**Table 2:** Enzymatic activity of combined complex I and complex III (CI+CIII) and citrate synthase (CS) measured in thorax crude homogenates of drosophila, expressed in U.mg^-1^ proteins.

	15 days old		25 days old	
	**CI+CIII**	**CS**	**CI+CIII**	**CS**
(*ore*);*OreR*	157.4 ± 9.5^a^	0.65 ± 0.17^a^	161.1 ± 9.4^a^	0.68 ± 0.038^a^
(*ore*);*Aut*	158.9 ± 14.2^a^	0.70 ± 0.027^a^	173.0 ± 9.8^a^	0.63 ± 0.029^a,b^
(*simw^501^*);*Aut*	151.4 ± 11.0^a^	0.61 ± 0.019^a^	165.5 ± 12.9^a^	0.61 ± 0.041^a,b^
(*simw^501^*);*OreR*	89.7 ± 6.56^b^	0.43 ± 0.023^b^	102.4 ± 5.26^b^	0.52 ± 0.028^b^

*Letters (^*a,b*^) denote significant differences between genotypes at either 15 or 25 days old.*

### Markers of Oxidative Stress and H_2_O_2_ Production

Oxidative stress was assessed by the fluorescent marker CellROX^®^ Deep Red Reagent in muscle fibers (Figure 2C) and was only influenced by the MtDNA (Table 1). Although, a slight increase was detected in (*simw^501^*);*OreR* at both ages, this was not significantly different than the other genotypes (Figure 2D). As an alternate method, oxidative damages to proteins and lipids were also measured. While no effects on protein carbonyls were found, we observed significant effects of MtDNA, nuDNA, and Age on TBars, but no interaction effects (Table 1). The highest level of TBars was measured in (*simw^501^*);*OreR* at 25 days old with 23.19 ± 0.84 μmol.mg^-1^ proteins, but it was not significantly different from the other genotypes at the same age [19.26 ± 1.31, 18.44 ± 1.58, and 19.43 ± 1.10 μmol.mg^-1^ proteins for (*ore*);*OreR*, (*ore*);*Aut*, and (*simw^501^*);*Aut*, respectively].

**FIGURE 3:**
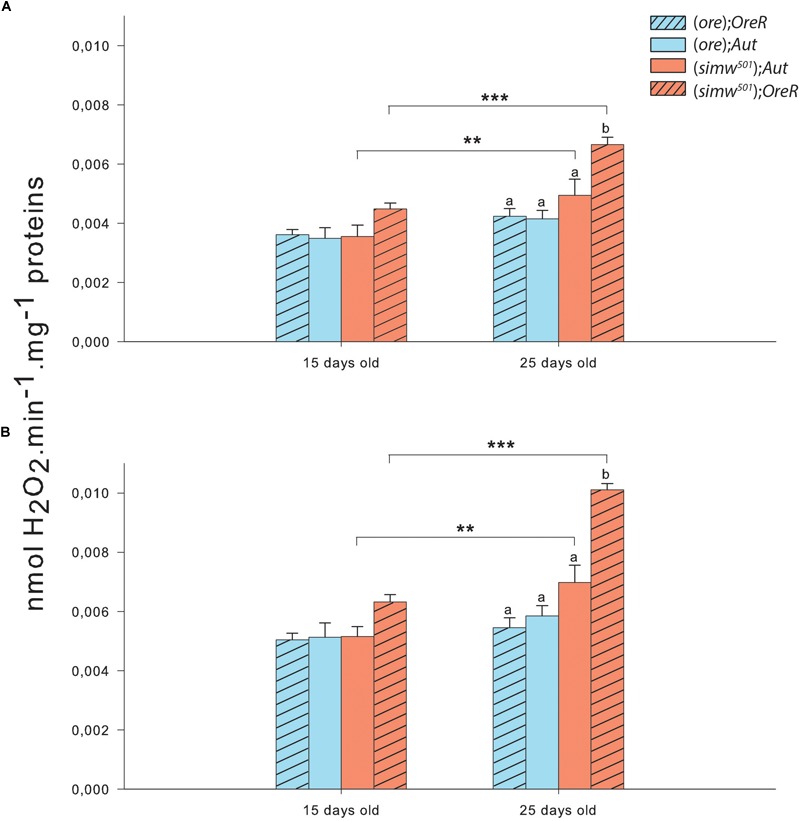
Hydrogen peroxide production of isolated mitochondria from thorax of *Drosophila*. **(A)** Hydrogen peroxide production measured in presence of pyruvate+malate+proline+ADP+glycerol-3-phosphate+rotenone triggering the maximum ROS production by complex I. **(B)** Hydrogen peroxide production measured in presence of pyruvate+malate+proline+ADP+glycerol-3-phosphate+rotenone+antimycin A triggering the maximum ROS production by complexes I and III. Results are presented as means ± s.e.m. of nmol H_2_O_2_ produced per minute per mg of proteins. ^∗^ denote differences (*post hoc* Tukey’s test) between ages with ^∗^*P* < 0.05, ^∗∗^*P* < 0.01, and ^∗∗∗^*P* < 0.001. Letters denote differences between the four genotype combinations.

As ROS have been shown to be a potential messenger triggering a nuclear response to mitochondrial dysfunctions, we also evaluated the H_2_O_2_ production derived from the production of superoxide anion in isolated mitochondria. Both conditions tested, i.e., maximal H_2_O_2_ production at the level of complex I and at the level of complexes I + III, displayed the same pattern i.e., effects of MtDNA, NuDNA, Age as well as of the interactions MtDNA × NuDNA and MtDNA × Age (Table 1). Moreover, maximal H_2_O_2_ production at the level of complexes I + III was also influenced by the triple interaction MtDNA × NuDNA × Age (Table 1). Specifically, H_2_O_2_ production increased at 25 days for (*simw^501^*);*Aut* and (*simw^501^*);*OreR* (*P* = 0.007 and *P* < 0.001 at the level of complex I; *P* = 0.008 and *P* < 0.001 at the level of complexes I + III; Figure 3). Additionally, in 25 days old (*simw^501^*);*OreR*, H_2_O_2_ production was significantly higher compared to the other mitonuclear genotypes (Figure 3A,B), which is in accordance with our measurements with the CellROX^®^ marker and the TBars assay.

### Mitochondrial Oxygen Consumption and Mitochondrial Complexes Activity

**FIGURE 4:**
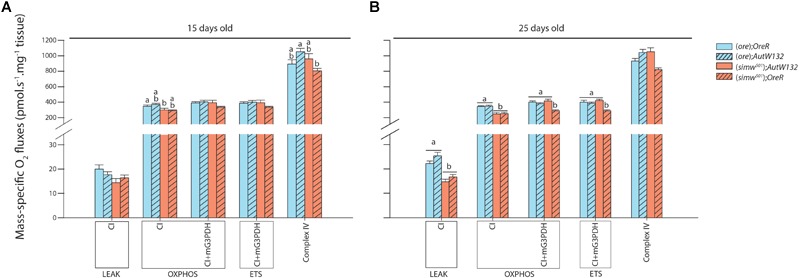
Mitochondrial respiration measured in permeabilized thorax of *Drosophila*. **(A)** *Drosophila* sampled at 15 days old. **(B)** *Drosophila* sampled at 25 days old. Results are expressed as pmol O_2_ consumed per second per mg of fibers. Mitochondrial functions are presented with the abbreviation(s) of the complex(es) involved followed by the state of respiration (complex-STATE) and were measured in presence of pyruvate+malate+proline+ADP (CI-OXPHOS); +glycerol-3-phosphate (CI+mG3PDH-OXPHOS); +FCCP (CI+mG3PDH-ETS); +rotenone and antimycin A used to correct for residual O_2_ consumption; +TMPD+ascorbate (Complex IV). Results are means ± s.e.m. Letters denote differences between the four genotype combinations.

Mitochondrial oxygen consumption was investigated in permeabilized thoraces of drosophila after different substrate combinations. All preparations assessed showed well-coupled respiration denoted by high P/L which was only influenced by Age (Table 1), as well as good integrity of mitochondrial outer membrane (small effect of exogenous cytochrome c on oxygen consumption, results not shown). The proton leak at the level of complex I (CI-LEAK) was influenced by MtDNA, Age and the interaction MtDNA × Age (Table 1). At 15 days old, no significant differences were detected in CI-LEAK (Figure 4A). At 25 days old, CI-LEAK was, however, lower for (*simw^501^*);*Aut* compared to the genotypes harboring the (*ore*) mtDNA (*P* < 0.001 and *P* = 0.02 for (*ore*);*Aut* and (*ore*);*OreR*, respectively) and (*simw^501^*);*OreR* also presented lower CI-LEAK than (*simw^501^*);*Aut* (*P* = 0.005; Figure 4B). For the other respiration rates, only (*simw^501^*);*OreR* presented significantly lower respiration rates than (*ore)*;*Aut* for CI-OXPHOS (*P* = 0.02) and Complex IV (*P* = 0.03) when measured at 15 days old (Figure 4A).

Interestingly, at 25 days old, (*simw^501^*);*Aut* and (*simw^501^*);*OreR* presented significantly lower CI-OXPHOS than (*ore*);*Aut* and (*ore*);*OreR* (all *P*-values ≤ 0.001, Figure 4B). Following addition of glycerol-3-phosphate as well as FCCP, (*simw^501^*);*OreR* was still lower but not (*simw^501^*);*Aut* (CI+mG3PDH-OXPHOS and CI+mG3PDH-ETS, Figure 4B). We next measured the catalytic capacity of CI+CIII to see if the depressed mitochondrial respiration rates observed when the mitochondrial tRNA mutation is present are related to the capacity of the ETS to reduce NADH. Consistently with respiration rates, the enzymatic activity of CI+CIII was lower in (*simw^501^*);*OreR* than in the other lines at both ages (Table 2).

## Discussion

In this study, we have thoroughly analyzed mitochondrial phenotypes harboring different mitonuclear combinations in flies at two different ages. Our results show that: (i) at 25 days old, a mitochondrial tRNA mutation expressed in two different nuclear backgrounds [combinations (*simw^501^*);*Aut* and (*simw^501^*);*OreR*] causes a decrease in oxygen consumption when mitochondrial respiration is supported by electron entrance at the level of complex I; (ii) at the same age, this decrease is alleviated upstream of complex III by an increase in respiration rate owing to the nuclear encoded mG3PDH in the mitonuclear combination (*simw^501^*);*Aut*; (iii) in the (*simw^501^*);*OreR* which harbors the incompatibility, a strong increase in mitochondrial DNA copy number was observed (a process likely triggered by an observed increase in H_2_O_2_ production), leading to mtDNA excess which could indicate replication errors. We also found that aging has more effects on the genotypes with the (*simw^501^*) mtDNA than those with the (*ore*) as shown by an increase in maximal H_2_O_2_ production by complex I and complexes I and III, and a decrease of CI-OXPHOS states. Specifically, the (*simw^501^*);*OreR* genotype is affected at an early age, and Drosophila with the (*simw^501^*);*Aut* genotype are more affected in terms of H_2_O_2_ production at the level of complex I when measured at 25 days old compared to 15 days old.

Although not naturally occurring, our model allows us to determine the contribution of mtDNA, nuDNA and mitonuclear interactions at two different biological ages and the importance of SNPs occurrence at the level of mitochondrial tRNA when expressed in a nuclear background carrying either a compatible [in (*simw^501^*);*Aut*] or an incompatible [in (*simw^501^*);*OreR*] associated amino-acyl-tRNA synthetase. The decreased CI+CIII enzymatic activity and respiration rates observed in (*simw^501^*);*OreR* flies at both ages but not in the other combinations tested seems to be a major consequence of the incompatibility between (*simw^501^*) mitochondrial tRNA^tyr^ and *OreR* tyrosine-tRNA synthetase. This result is consistent with other studies showing decreased enzymatic activities of individual ETS complexes encoded by both genomes (CI, CIII, and CIV) in (*simw^501^*);*OreR* and suggests an impairment of mitochondrial functions that leads to major consequences at the phenotypic level (Meiklejohn et al., 2013; Holmbeck et al., 2015).

We found that at 25 days old, (*simw^501^*);*Aut* presented reduced mitochondrial oxygen consumption when assayed with complex I substrates (CI-LEAK and CI-OXPHOS) to the same extent as (*simw^501^*);*OreR.* It suggests that the mitochondrial tRNA^tyr^ SNP and therefore the mitochondrial genome has an impact on oxygen consumption by mitochondria via the input of electrons through the ETS, but that this effect is more important in 25 days old flies. Considering that complex I has the highest number of mitochondrial subunits of all ETS complexes (7 subunits), our results suggest major consequences of any impairment in mtDNA translation and transduction of mitonuclear exchanges on functional properties of complex I. Morevover, the depressed CI mitochondrial functions found in 25 days old (*simw^501^*);*Aut* suggest a high sensitivity to mutation accumulation with aging in the mitochondrial genes encoding this complex. This is clearly illustrated by the strong impact of the MtDNA and Age factors in the statistical model at the level of mitochondrial respiration when electrons are exclusively supplied to complex I. Of relevance, complex I also contains the most tyrosines (98) which implicates a major effect of the tRNA-synthetase interaction on the mitochondrial functions (Holmbeck et al., 2015). Surprisingly, it is when the ETS is saturated from convergent electron flow (complex I and mG3PDH) that the mitochondrial phenotype is mostly affected by nuclear DNA (Table 1).

Increasing the electron flux in the ETS by supplying G3P (CI+mG3PDH-OXPHOS) resulted in a complete compensation in 25 days old (*simw^501^*);*Aut*, with respiration rates reaching the same level as (*ore);Aut* and (*ore*);*OreR* of the same age. Although the mitochondrial tRNA SNP is linked to OXPHOS deficiencies, (*simw^501^*);*Aut* flies are able to bypass these deficiencies when higher ETS capacity is mobilized upstream of complex III via mG3PDH activity. The mG3PDH is at the crossroads of glycolysis, oxidative phosphorylation, and fatty acid metabolism, and is fundamental for NADH homeostasis in the cytosol (Mráček et al., 2013; Orr et al., 2014; McDonald et al., 2018). Therefore, the mG3PDH represents a plausible alternative for mitochondria to alleviate a complex I deficiency and maintain respiratory functions. Thus, our results reveal flexibility of mitochondria that can remodel the pathways of electron supply into the ETS, at least when complex I is partly compromised.

We found an almost three fold increase in the mtDNA copy number in (*simw^501^*);*OreR* at both ages, suggesting a strong response at the level of mtDNA regulation. This could reflect an increase of mitochondrial biogenesis, and thus of mitochondrial content. Indeed, increased mtDNA copy number is usually associated with mitochondrial biogenesis (Lee and Wei, 2005; Clay Montier et al., 2009; Picca and Lezza, 2015). Moreover, increased mitochondrial biogenesis has been shown to compensate for mitochondrial dysfunctions, and stimulating this process can be beneficial for some mitochondrial related diseases (Moreno-Loshuertos et al., 2006, 2011; López-Lluch et al., 2008; Chandra et al., 2016). Despite the mitochondrial dysfunctions detected in (*simw^501^*);*OreR*, we did not detect evidence for increased mitochondrial biogenesis, as mitochondrial content remains significantly lower than in the other genotypes (as observed with mitochondrial labeling by Mitotracker^®^ and CS activity), highly contrasting with the results on relative mtDNA copy number. The strong response in mtDNA seems to be a consequence of the mitochondrial dysfunctions that possibly participate in mutation accumulation, leading to the observed phenotype (DeBalsi et al., 2017; Kauppila et al., 2017). Indeed, mtDNA proliferation has been reported as a compensatory effect of mtDNA deletions (Bai and Wong, 2005; Clay Montier et al., 2009) and that this loss of control is associated to aging and to either nuclear or mtDNA mutations leading to mitochondrial dysfunctions (Clay Montier et al., 2009). Concomitant with this increased mtDNA copy number, we observed a slight increase in ROS content and in maximal H_2_O_2_ production at 15 days old, as well as a significant increase in maximal H_2_O_2_ production at 25 days in the same mitonuclear genotype combination. It therefore suggests that mitochondria from (*simw^501^*);*OreR* are producing more ROS than the other genotypes and that the genotypes harboring the (*simw^501^*) mitochondrial DNA have increased ROS production with aging. However, this increased ROS production did not translate into oxidative damages to proteins or lipids. An explanation for these results would be that the carbonyl and TBars assays used in our study are not sensitive enough to detect oxidative damages to proteins and lipids, respectively, and that other markers such as thiol and 4-hydroxynonenal assays might be worth to measure as an alternative. However, (*simw^501^*);*OreR* flies displayed slightly higher TBars content when both ages were combined [17.41 ± 0.86, 16.24 ± 1.11, 16.81 ± 1.03, and 20.97 ± 1.04 for (*ore*);*OreR*, (*ore*);*Aut*, (*simw^501^*);*Aut* and (*simw^501^*);*OreR*, respectively], suggesting slightly higher oxidative stress in this genotype.

It is surprising that mitochondrial content was lower in (*simw^501^*);*OreR* despite increased mtDNA copy number, which is usually associated with mitochondrial biogenesis (Lee and Wei, 2005; Clay Montier et al., 2009; Picca and Lezza, 2015). Simultaneously with an increase of mitochondrial biogenesis, mitochondrial dysfunctions may trigger mitophagy (Palikaras and Tavernarakis, 2014; Palikaras et al., 2015). These two opposing but tightly coordinated processes are essential for cellular adaptation in response to metabolic state, stress and other intracellular or environmental signals (Palikaras and Tavernarakis, 2014). It is therefore possible that the lower mitochondrial content observed in (*simw^501^*);*OreR* is due to mitophagy rather than to an impairment of biogenesis. Indeed, our results show similar pattern (increase in mtDNA copy number concomitant with a decrease in mitochondrial content) to what has been observed in mitochondrial encephalomyopathy, lactic acidosis, and stroke-like episodes (MELAS) fibroblasts, which is a disorder caused by mutations in mitochondrial tRNA genes (Cotán et al., 2011). This is also consistent with the locomotor defects observed in (*simw^501^*);*OreR* as well as structural mitochondrial abnormalities (loose cristae structure and matrix gaps) compared to the other genotypes detected by Holmbeck et al. (2015). Indeed, it has been suggested that this mitonuclear genotype provides a good model for the study of mitochondrial diseases associated with exercise intolerance in humans (Holmbeck et al., 2015). Measuring expressions of key genes involved in mitochondrial biogenesis as well as master regulators of mitophagy could bring insights on these mechanisms.

## Conclusion

Our investigation of the mechanisms of mitonuclear interactions shows that mitochondrial tRNA mutations affect several aspects of the metabolic phenotype, especially under aging, and that complex cellular process may occur during breakdown of co-adapted genes due to different compensatory mechanisms that can be of importance for genome co-evolution. We found that for the (*simw^501^*);*Aut*, the mG3PDH which is a nuclear-encoded complex of the ETS can participate in the maintenance of mitochondrial oxidative capacities by compensating for a complex I deficiency. When a mutation in the corresponding nuclear amino-acyl-tRNA synthetase occurs, as in the (*simw^501^*);*OreR* genotype, a mitonuclear mismatch appears. This results in a reduced catalytic capacity at the level of CI+CIII and diminished respiration rates likely leading to a more reduced state of ETS upstream of complex IV and inflation of maximal H_2_O_2_ production. This response can promote the strong increase observed in mtDNA copy number. These two concomitant responses (higher ROS production and mtDNA excess) could increase mutation accumulation that, in turns, exacerbate mitochondrial dysfunctions. Introgressed hybrids can however survive mitonuclear mismatch, even if growth and lifespan are clearly compromised (Zhu et al., 2014), bringing new insights into the evolutionary potential of hybridization. These findings provide important information to refine hypotheses concerning processes and patterns of mitonuclear genome co-evolution. For example, they suggest that complex I mitochondrial encoded genes could be important targets of positive selection associated to ROS metabolism and regulation as well as an important site for the control of aging.

The role of ROS in the aging process is still debated, mostly in short-lived invertebrates such as drosophila and nematodes, but our results confirm that the reduction in average lifespan observed in the (*simw^501^*);*OreR* genotype (Zhu et al., 2014) is clearly associated with a significant boost in mitochondrial H_2_O_2_ efflux when compared to other lineages. To which extent this increase in H_2_O_2_ production leads to oxidative stress that could be the cause of lifespan reduction remains to be tested but warrants further scrutiny. It is frequently postulated that proper epistasis between mitochondrial and nuclear genomes should be under strong selection because of the importance of mitochondria on cellular bioenergetics and of ROS metabolism on developmental and age-related processes such as apoptosis and inflammation (Lane, 2011; Horan et al., 2013; Wolff et al., 2014). Our results suggest that drosophila can cope with deficient ETS and overproduction of ROS, at least under optimal laboratory conditions. These genotypes offer great opportunity to assess how the compromised mitochondrial phenotype can survive to a more normal and stressful environment and therefore to which extent proper mitochondrial phenotype is required to ensure fitness in natural conditions. For example, Buchanan et al. (2018) noticed that (*simw^501^*);*OreR* had lower survival under infection with a natural pathogen, with females experiencing immunity–fecundity tradeoffs. While the impact of defective ETS on ROS mitochondrial efflux was clear in our study, we have not been able to detect any associated trace of oxidative stress. This suggests that either the mechanisms involved in buffering ROS were adequate to prevent any damage or that our markers did not provide the necessary resolution to detect significant ROS injuries. In the TBars assay for example, thiobarbituric acid does not only react with the products of lipids peroxidation such as malondialdehyde and is at best a weak marker of ROS attack on lipids. Further studies using more sensitive techniques, such GC-MS, are needed to precisely monitor some specific products of lipid peroxidation such as 4-Hydroxynonenal or 4-Hydroxyhexenal.

Another limitation of our study is that we characterized the cellular response following mitonuclear mismatch in flies at only two different biological ages. The two chosen ages (15 and 25 days old) are however not usually considered as advanced in *D. melanogaster*, and mutation accumulation as well as their effects on mitochondrial and ROS metabolism should thus be more important at older ages in this model. Other studies evaluating the effects of this mitonuclear mismatch in older flies and at several biological ages could thus bring new understanding on the role of mitonuclear epistasis in aging. Despite this limitation, we have detected exacerbation of ROS efflux at 25 days old whereas the compensatory mechanisms of increased mtDNA copy number occured much earlier. It will thus be also important to follow, along with ROS generation and mitochondrial functions, mtDNA quality (oxidation and mutation accumulation) over a wider range of lifespan to detect key characters susceptible to set physiological and longevity limitation.

## Author Contributions

NP and PB designed the research. NP, GC, and RB performed the research. FD and PB provided the materials for experiments. DR provided the different genotypes. NP analyzed the data and wrote the first draft of the manuscript. All authors participated in the subsequent versions of the manuscript.

## Conflict of Interest Statement

The authors declare that the research was conducted in the absence of any commercial or financial relationships that could be construed as a potential conflict of interest. The handling Editor and reviewer NJ declared their involvement as co-editors in the Research Topic, and confirm the absence of any other collaboration.
